# Assessment of neuropsychological trajectories in longitudinal population-based studies of children

**DOI:** 10.1136/jech.2007.071530

**Published:** 2008-12-05

**Authors:** R F White, R Campbell, D Echeverria, S S Knox, P Janulewicz

**Affiliations:** 1 Department of Environmental Health, Boston University School of Public Health, Boston, Massachusetts, USA; 2 Department of Neurology, Boston University School of Medicine, Boston, Massachusetts, USA; 3 Community Health Program, Tufts University, Massachusetts, USA; 4 Battelle Memorial Institute, Columbus, Ohio, USA; 5 University of West Virginia, Morgantown, West Virginia, USA

## Abstract

This paper provides a strategy for the assessment of brain function in longitudinal cohort studies of children. The proposed strategy invokes both domain-specific and omnibus intelligence test approaches. In order to minimise testing burden and practice effects, the cohort is divided into four groups with one-quarter tested at 6-monthly intervals in the 0–2-year age range (at ages 6 months, 1.0, 1.5 and 2.0 years) and at annual intervals from ages 3–20 (one-quarter of the children at age 3, another at age 4, etc). This strategy allows investigation of cognitive development and of the relationship between environmental influences and development at each age. It also allows introduction of new domains of function when age-appropriate. As far as possible, tests are used that will provide a rich source of both longitudinal and cross-sectional data. The testing strategy allows the introduction of novel tests and new domains as well as piloting of tests when the test burden is relatively light. In addition to the recommended tests for each age and domain, alternative tests are described. Assessment methodology and knowledge about child cognitive development will change over the next 20 years, and strategies are suggested for altering the proposed test schedule as appropriate.

This paper summarises the process of reviewing and selecting outcome measures that assess cognitive abilities as indicators of the development of brain structure and function from the age of 6 months to 20 years and offers a strategy for their use in epidemiological studies. This strategy applies measures that are applicable in prospective designs and allow description of the natural development of cognitive skills while monitoring potential environmental influences (eg, diet, social, media, chemicals) on developmental trajectories.

This assessment plan assumes a comprehensive evaluation of dimensions of intellectual development that encompasses both appropriate aspects of cognition and detection of vulnerable domains of function at specific stages of development. The strategy proposed is significantly influenced by the neurocognitive and neuropsychological literature. It is especially appropriate for interpreting developmental cognitive outcome data with regard to brain-behaviour relationships.

An initial assessment issue involves resolving the tension between testing “general intelligence” and assessing neuropsychological functioning. In the former case, an intelligence quotient (IQ) is computed based on administration of standardised omnibus tests. In the latter, domain-specific scores are computed based on administration of specific tests. This issue has received considerable attention in the developmental neurotoxicology literature. IQ tests have been used extensively in the study of certain types of toxicant exposures (especially lead and polychlorinated biphenyls (PCBs)), and it has been argued that IQ tests are preferable to other kinds of tests for their “public health value”. This belief posits that individuals besides psychologists understand the meaning of such tests, and the results are therefore more likely to be taken seriously and acted upon by the public health community and society at large.

In contrast, domain-specific neuropsychological tests have received more attention in recent years in behavioural toxicology because of their sensitivity to prenatal exposure to toxicants such as methylmercury^1^ and nicotine.^2^ In addition, it has been argued that these tests provide more insight into the underlying central nervous system (CNS) damage that may be associated with exposures, since there is a significant literature that links impaired performance within individual domains or patterns of impaired and intact performance across domains to specific types of brain damage (structural, neural system, neurotransmitter).

Epidemiological designs can allow consideration of both IQ and domain-specific outcomes at critical stages of vulnerability during child development. Such an approach provides the advantages of both types of assessment approaches as well as the potential to re-evaluate the results of existing studies when new data are acquired.

## DOMAINS OF RESEARCH INTEREST

Categorising the dimensions of cognition into component parts is challenging even to cognitive psychologists and neuropsychologists, who often divide or subdivide these dimensions in different ways. It is even more difficult to categorise existing cognitive and behavioural tests since few were designed to be pure tests of a specific aspect of cognitive processing or a single domain. For the purposes of developing a recommended battery, domains were identified using the labels commonly applied in clinical neuropsychology.^3^ ^4^ In addition, careful consideration was given to key aspects of processing that should be assessed within each domain. The following list identifies the domains selected by the authors and associated defining characteristics. The definitions are not meant to be exhaustive but to give the reader an overview of the types of skills subsumed under each domain and assessed by associated neuropsychological tasks.

### General intelligence/mental abilities/omnibus cognitive skills tests

These tests consist of subtests with various labels purported to measure aspects of cognitive function. Subtest scores are summed in order to obtain overarching measures such as IQ, often accompanied by omnibus measures of verbal abilities (eg, Verbal IQ, Verbal Comprehension Index), visual-motor or visuospatial skills (eg, Performance IQ, Perceptual Organization Index), attention/working memory (eg, Working Memory Index, Attention Index) or speed of processing (eg, Processing Speed Index).

### Academic skills

This domain includes skills such as reading words or paragraphs, spelling and completing arithmetic problems.

### Attention

This domain encompasses several processes including the capacity to focus on and attend to stimuli over a period of time (sustained attention, often assessed by Continuous Performance Tests) and the capacity to take in and report back stimuli immediately after presentation (eg, Forward Digit Span or Visual Pointing Span).

### Executive function/working memory

This is a complex domain that historically includes the capacities to learn and manipulate stimuli (eg, Digit Span Backward, Visual Pointing Span Backward), to invoke strategies for manipulating novel stimuli (any task with a structure that enhances task completion if recognised) or to solve novel problems (problem solving tests). This domain includes skills such as the ability to acquire the “set” of new tasks and to maintain the set of the task while completing it as well as the ability to flexibly switch from one set of task requirements to another. Inhibition of dominant or distracting stimuli in order to attend to critical stimuli is also included in this domain.

### Language/verbal skills

This domain includes basic linguistic abilities such as the capacity to produce phonemes, lexical development and production of words, speech comprehension and linguistic aspects of writing and reading. Language skills are often divided into *expressive* and *receptive* components. Applied verbal skills, such as vocabulary definitions, are sometimes included in this domain.

### Visuospatial abilities

These non-verbal abilities generally invoke the processing and manipulation of visual designs, the spatial or physical aspects of environmental objects or constructional skills. These abilities are assessed by tasks such as drawing designs, recognising objects presented in degraded form or embedded in a more complex visual array, or assembling puzzles or block designs. Constructional tasks involve motor output, but there are visuospatial tasks that require simply the mental manipulation of spatial information (eg, identifying the correct outline of an object presented in cut-up form, matching faces, matching angles).

### Learning and memory

This domain encompasses several aspects of memory function. *Declarative memory* is generally divided into anterograde and retrograde memory function. *Anterograde memory* refers to the learning of new information, retention of information over shorter and longer delays, and the capacity for retrieval of information from memory stores. It can be assessed using both recall and recognition paradigms (recall paradigms get at the individual’s capacity to retrieve information at will while recognition paradigms are often better at assessing capacity for learning and retention when retrieval problems exist). Anterograde memory functions are sometimes divided into verbal and visuospatial components, generally associated with dominant and non-dominant memory function, although visuospatial memory skills are also frequently affected in individuals with basal ganglia and white matter dysfunction. Anterograde memory is measured in many ways including the presentation of stories, lists of words, designs or objects for immediate learning, with delayed recall and recognition (multiple choice) conditions. *Retrograde memory* refers to the capacity to remember events or information from earlier stages of the individual’s life. It can be tested using famous faces, questions about historical events or facts, or questions about the individual’s personal history. *Procedural learning and memory* refers to the individual’s capacity to learn and remember a problem-solving sequence (eg, reading words in a mirror) or a motor skill (eg, driving a car).

### Motor skills

These abilities refer to the individual’s capacity to carry out manual motor activities. Using neuropsychological tests, they are generally assessed using the hands (manual motor dexterity), with evaluation of speed and accuracy. Tasks may be relatively simple (tapping a computer key or finger tapping apparatus), complex and requiring coordination as well as speed (pegboard tasks) or integrative (writing or typing symbols to match digits on a coding task).

Other domains can be included if relevant or if standardised tests become available. These include expressive and receptive prosody, motivation/malingering and tactile/kinesthetic function. Tasks assessing the above domains and conditions under which they might be applied are discussed in this paper.

## METHODS

The development of a recommended longitudinal cognitive assessment strategy consisted of several steps. First, a decision was made to focus on quantitative measures that detect subtle preclinical cognitive dysfunction. Second, it was decided to expand criteria for test selection beyond outcomes that solely depend on clinical diagnosis of neuropsychiatric disorders, as this strategy could miss more subtle dysfunction in children at critical ages and has little power to describe cognitive development. Further, the list of critical domains and the stages of development that should be evaluated required definition. Using this framework, the list of tests and test batteries that evaluate these domains was developed and the tests were then reviewed according to a set of criteria.

Throughout this process, existing clinical and scientific knowledge about child development was supplemented with literature on developmental neurotoxicology, which describes the relationships between exposure to common environmental chemicals and their effects on brain function. Studies in this field have taken advantage of the power of cognitive developmental tests as measures of brain function and have thus produced important information on domains of cognitive function that are especially vulnerable to the subtle effects of environmental influences, ages at which particular vulnerabilities may appear in specific functional domains, effect sizes of subtle deficits attributable to environmental influences and test instruments that are especially useful to detect subtle cognitive deficits in children.

Based on a review of the literature and on specific criteria developed for test selection, a subset of tests was identified and further explored for inclusion in a proposed assessment battery. In addition, strategies for assessing children at critical stages during development were considered. It should be noted that the development of a battery to be used over long periods of time relies on the state of the art in the field at present. Therefore, one must anticipate that tests and test strategies will be adjusted at intervals as long-term studies progress.

### Overview of tests and test batteries available

Many cognitive and neuropsychological tests have been published that directly or indirectly assess the domains described above. A compendium that was too extensive to publish with this paper is available on the NICHD website (http://www.nationalchildrensstudy.gov/research/reviewsreports/Pages/Neuropsychological-Assessments-in-Children-from-a-Longitudinal-Perspective-for-the-National-Children-s-Study.pdf). It includes approximately 135 tests and scales that are listed by domains of research interest and include applicable age ranges. Included in that table are five of the most commonly used batteries that have been recommended for evaluating children and adults with suspected or known exposure to chemical toxicants. The listings are not exhaustive but include all tests considered for inclusion in the recommended battery. Some other types of novel tasks and tests are mentioned in this paper even though they do not exist as published standardised tests.

### Criteria used in test review

The tests listed in the compendium were reviewed by RFW and a shorter list of tests was selected as candidate screening tasks. Criteria for test selection are discussed below.

#### Place of test in child development literature

Tests were considered with regard to their place in the field of developmental psychology. Tests that have been in widespread use by clinicians and researchers were given preference. This was done for several reasons. First, such tests are generally feasible with regard to administration. Second, they are more interpretable because they are associated with more published information concerning the relationship of test outcomes to particular types of developmental disorders (eg, attention, learning disabilities, speech and language disorders, extremes in IQ, motor deficits), neurological diseases (eg, epilepsy, brain tumours, traumatic brain injury), neuropsychiatric disorders (eg, autism, childhood depression, personality disorders, post-traumatic stress disorder, anxiety), medications and medical conditions (eg, genetic disorders affecting cognition, metabolic disorders, respiratory diseases). Such information contributes to the capacity of the tests to assist in screening (for triaging children on to other evaluations) at the same time they serve as outcome measures. In addition, structure-function relationships have been described for many of the tests, relating impaired performance on certain tests (or patterns of impaired and retained performance on groups of tests) to particular structures of the CNS. This knowledge is critical in that it may allow investigators to form hypotheses concerning the structural or functional elements of the CNS that may be affected by exposures. These hypotheses can serve as the basis for further investigations (eg, sophisticated neuroimaging).

#### Place of test in the neurotoxicant literature

If tests had proven sensitivity to low level and subtle effects of chemical exposures, they were high on the candidate list for exploring exposure-outcome relationships in a large epidemiological screening study where toxicant exposure is being measured. They also may have value in examining the subtle effects of other types of exposure (eg, stress, violence, medications, drugs, stimulus deprivation or overstimulation, undernourishment/overnourishment/malnourishment). The website cited above includes extensive tables that summarise the developmental neurotoxicology literature that included standardised test outcomes.

#### Construct validity

Priority was given to tests that have demonstrated ability to assess specific domains. In addition, an attempt was made to identify tests that could reliably assess a specific cognitive process (or a set of processes) within a domain, and it was also deemed necessary to balance the types of tests used within and across domains at specific ages.

#### Demographics

Preference was given to tests for which the effects of age (in months at the younger ages) and gender have been defined/quantified. It was critical to include tests with a wide age range since longitudinal studies may follow individuals from birth to age 20. Effects of parental education and intelligence were also considered, if available.

#### Culture/ethnicity/language

Available data on the relationships between culture and language and test performance were also considered. Are there ethnicity/cultural effects on test performance, and if so, what are they? Are there special versions of the tests for children from specific subcultures? How “culture-fair” is the test? Also considered were effects of primary languages and multilingualism on test performance, including availability of the tests in languages other than English. Information was not available on these variables for many of the tests, but tests with such information received special consideration.

#### Psychometrics

Sensitivity to subtle effects of exposures requires tests that possess certain psychometric characteristics. These include a sufficient range of outcome scores and variance to reliably identify exposure-outcome relationships. They must also be reliable (especially with regard to test-retest reliability), that is, result in similar scores in the same person when measured at repeated time points with no change in exposure. It is also important that they have demonstrated validity with regard to the construct they are measuring as demonstrated by their relation to other known tests (see above). Priority was also given to tests that are well standardised. Availability of appropriate normative values for test performance at different ages and for other variables was considered to be important for certain purposes (eg, characterising cohort performance relative to the US population). However, for data analysis purposes, raw scores are usually the outcome of choice in epidemiological studies.

#### Other factors

Other test characteristics that were considered important include ease of administration, acceptability to children, acceptability to parents, reasonable difficulty levels without extreme frustration for most children, efficiency and capacity of the test to contribute to screening/triaging. Participant burden is a major issue and even outstanding measures were discarded if it they are too time-consuming to allow inclusion of other required measures. Finally, it must be stated that RFW’s experience using the tests in research and clinical settings played a role in the test review. Batteries were limited in administration time to 1–2.5 h, depending on age.

### Battery design and testing strategy for prospective developmental assessments

The literature on exposure-outcome relationships is incomplete with regard to data on age at exposure and age at which exposure effects can be detected. For example, the neurotoxicology literature suggests that lead exposure in early childhood is associated with IQ changes^5^ and that prenatal methylmercury exposure is associated with domain-specific neuropsychological effects at the age of 7 years.^6^ However, systematic studies across ages of exposure, ages at which outcomes are measured and specific toxicants do not yet exist. It is therefore difficult to pinpoint critical ages at which specific types of neuropsychological outcomes should be measured.

Given the existing knowledge, it appears that the optimal strategy is to acquire outcome data at as many ages as possible. Because practice effects are large and can overwhelm subtle exposure effects, it is not recommended that each child be tested every year. A strategy that would allow the collection of data in yearly age increments but prevent practice effects is to divide a large cohort into groups of children. Testing each child every 4 years beginning at age 3–6 would result in neuropsychological outcome data on a large group of children at each age. Before age 3, it is recommended that the four groups of children be tested at 6 months, 1 year, 1.5 years or 2 years. This strategy is outlined in table 1, which gives an age × domain × test representation of a recommended battery. Each of the four subgroups of children is designated by the letters A, B, C and D. This strategy also allows repeat testing with certain key tasks at widely divergent ages, facilitating longitudinal follow-up on exposure-outcome relationships.

**Table 1:** Recommended test matrix for a cohort of 100 000 children with 25% in each of four age exposure groups (A, B, C, D)

Age at testing	Battery length (min)	Group(s) to be tested	Tests
6 months	20	A	Screening domain: Bayley-II
			
1 year	20	B	Screening domain: Bayley-II
			
1.5 years	30	C	Screening domain: Bayley-II
			
2 years	30	D	Screening domain: Bayley-II
			
3 years	60	A	IQ domain: WPPSI-III (block design, matrix reasoning, vocabulary subtests)
			Attention domain: Conners Rating Scale-R
			Language domain: EVT (naming subtest); PPVT-III
			Visuospatial domain: Beery Visual-Motor Integration Test-5
			Motor domain: Revised Purdue Pegboard Novel/pilot tests (group C)
			
4 years	75	B	IQ domain: WPPSI-III (block design, matrix reasoning, vocabulary subtests)
			Attention domain: Conners Rating Scale-R; Conners CPT-II
			Language domain: EVT (naming subtest); PPVT-III
			Visuospatial domain: Beery Visual-Motor Integration Test-5
			Learning and memory domain: Coding recall (Wechsler test)
			Motor domain: Revised Purdue Pegboard; Coding subtest (Wechsler test) Novel/pilot tests (group D)
			
5 years	95	C	IQ domain: WPPSI-III (block design, matrix reasoning, vocabulary subtests)
			Attention domain: Conners Rating Scale-R; Conners CPT-II
			Language domain: EVT (naming subtest); PPVT-III
			Visuospatial domain: Beery Visual-Motor Integration Test-5
			Learning and memory domain: CVLT-Children’s edition; WRAML-2 (stories subtest) Coding recall (Wechsler test)
			Motor domain: Revised Purdue Pegboard; Coding subtest (Wechsler test) Novel/pilot tests (group A)
			
6 years	95	D	IQ domain: WPPSI-III (block design, matrix reasoning, vocabulary subtests)
			Attention domain: Conners Rating Scale-R; Conners CPT-II
			Language domain: BNT; PPVT-III
			Visuospatial domain: Beery Visual-Motor Integration Test-5
			Learning and memory domain: CVLT-Children’s edition; WRAML-2 (stories subtest); Coding recall (Wechsler test)
			Motor domain: Revised Purdue Pegboard; Coding subtest (Wechsler test) Novel/pilot tests (group B)
			
7 years8 years9 years10 years	145145150150	ABCD	IQ domain: WASI (block design, matrix reasoning, vocabulary, similarities subtests)
			Academics: Wide Range Achievement Test 3 (spelling, arithmetic, reading subtests)
			Attention domain: Conners Rating Scale-R; Conners CPT-II; Digit Span Forwards (Wechsler test)
			Executive function domain: WCST
			Language domain: BNT; PPVT-III
			Visuospatial domain: Bender Gestalt-II
			Learning and memory domain: Bender Gestalt-II (recall condition); CVLT-Children’s edition; WRAML-2 (stories subtest)* stimuli changes at age 9; Coding recall (Wechsler test)
			Motor domain: Grooved Pegboard; Finger Tapping Test; Coding subtest (Wechsler test) Novel/pilot tests (7 years group C; 8 years group D; 9 years group A; 10 years group B)
			
11 years12 years13 years	115115115	ABC	Attention domain: Conners’ CPT-II ; Digit Span Forwards (Wechsler test)
			Executive function domain: Digits Backwards (Wechsler test); TMT
			Language domain: EVT; CELF-4 (sentence structure subtest)
			Visuospatial domain: HVOT; ROCF; Berry Visual Motor Integration Test-5
			Learning and memory domain: ROCF (recall condition); WRAML-2 (stories and verbal learning subtests); Coding recall (Wechsler test)
			Motor domain: Grooved Pegboard; Finger Tapping Test; Coding subtest (Wechsler test)Novel/pilot tests (11 years group C; 12 years group D; 13 years group A
			
14 years	120	D	Attention domain: Conners Rating Scale-R; Conners CPT-II; Digit Span Forwards (Wechsler test)
			Executive function domain: Digits Backwards (Wechsler test); TMT
			Language domain: EVT; CELF-3 (sentence structure subtest)
			Visuospatial domain: HVOT; RCOF; Berry Visual-Motor Integration Test-5
			Learning and memory domain: ROCF (recall condition); WRAML-2 (stories and verbal learning subtests); Coding recall (Wechsler test)
			Motor domain: Grooved Pegboard; Finger Tapping Test; Coding subtest (Wechsler test) Novel/pilot tests (group B)
			
15 years16 years	160160	AB	IQ domain: WASI (block design, matrix reasoning, vocabulary, similarities subtests)
			Academics: Wide Range Achievement Test 4 (spelling, arithmetic, reading subtests)
			Attention domain: Conners Rating Scale-R; Conners CPT-II; Digit Span Forwards (Wechsler)
			Executive function domain: Digits Backwards (Wechsler); WCST
			Language domain: BNT; PPVT-III
			Visuospatial domain: Bender Gestalt-II
			Learning and memory domain: Bender Gestalt-II (recall condition); CVLT-II; WRAML-2 (stories subtest); Coding recall (Wechsler)
			Motor domain: Grooved Pegboard; Finger Tapping Test; Coding subtest (Wechsler) Novel/pilot tests (15 years group C; 16 years group D)
			
17 years18 years	155150	CD	IQ domain: WASI (block design, matrix reasoning, vocabulary, similarities subtests)
			Academics: Wide Range Achievement Test 4 (spelling, arithmetic, reading subtests)
			Attention domain: Conners Rating Scale-R (not at age 18); Conners CPT-II; Digit Span Forwards (Wechsler test)
			Executive function domain: Digits Backwards (Wechsler test); WCST
			Language domain: BNT, PPVT-III
			Visuospatial domain: Bender Gestalt-II
			Learning and memory domain: Bender Gestalt-II (recall condition); CVLT-II; Wechsler Memory Scale-III (logical memory subtest); Coding recall (Wechsler test)
			Motor domain: Grooved Pegboard; Finger Tapping Test; Coding subtest (Wechsler test) Novel/pilot tests (17 years group A; 18 years group B)
			
19 years20 years	8585	A and BC and D	Attention domain: Conners CPT-II
			Executive function domain: TMT
			Language domain: EVT; CELF-4 (sentence structure subtest)
			Visuospatial domain: HVOT; ROCF
			Learning and memory domain: ROCF (recall condition); CVLT-II; Wechsler Memory Scale-III (logical memory subtest)
			Motor domain: Grooved Pegboard; Finger Tapping Test Novel/pilot tests (19 years group A and B; 20 years group C and D)

BNT, Boston Naming Test; CELF, Clinical Evaluation of Language Fundamentals; CVLT, California Verbal Learning Test; CPT, Conners Continuous Performance Test; EVT, Expressive Vocabulary Test; HVOT, Hooper Visual Organization Test; PPVT, Peabody Picture Vocabulary Test; ROCF, Rey Osterrieth Complex Figure; TMT, Trail Making Test; WASI, Wechsler Abbreviated Scale of Intelligence; WCST, Wisconsin Card Sorting Test; WPPSI-III, Wechsler Preschool and Primary Scale of Intelligence; WRAML, Wide Range Assessment of Memory and Learning.

The testing strategy necessarily initiates testing of different domains at different ages. For example, executive function testing is not introduced until age 7. Testing of learning and memory is limited before age 7. These recommendations reflect the developmental curve of domain-specific skills as well as the availability of tests appropriate for certain ages. These factors are discussed in greater detail below.

The testing strategy allows for the introduction of different tests that assess the specific domains at different ages. Using this strategy, domain-specific findings observed on one test can be evaluated somewhat later with a similar test from the same domain, allowing a chance to evaluate convergent validity. It is also possible to examine more than one specific aspect of cognitive processing within each domain. In designing the battery, attempts have been made to evaluate parallel aspects of cognitive processing during each age range and within each domain.

Consideration was given to using the neuropsychological outcome measures as “triggers” prompting complete diagnostic evaluations in children who may have specific types of developmental disorders. Thus, the recommended battery incorporates the ages and criteria for using outcome measures to triage children into screening for mental retardation, disorders of attention and learning, motor coordination deficits, autism and neurological disorders.

Finally, the views presented here are guided by personal experience of RFW, which includes 30 years of work in research and clinical settings assessing individuals across the lifespan. This work has included prospective evaluation of children with environmental exposures during infancy and early childhood, cross-sectional research on environmental toxicant exposures in childhood, occupational exposure studies with adults and the long-term evaluation of neurodegenerative disorders in elderly subjects. In all of this research, neuropsychological test techniques have been applied as a method for uncovering the underlying neuropathological mechanisms of action for cognitive development. The battery described below is viewed as a starting point in planning cognitive and neuropsychological assessments of a large cohort at various ages.

## RESULTS

### Recommended neuropsychological outcome battery and alternative tests

Table 1 summarises the recommended test battery to be administered at each age level for each proposed domain. The age ranges cover 2 years at the lowest level (with 6-month intervals for testing until age 2) and 4 years after that until age 19–20 years (so that 25% of the cohort is tested at each age). A suggested testing schedule in a large study could therefore contain four groups: Group A: 25 000 children tested at ages 5, 3, 7, 11, 15 and 19 years of age ; Group B: 25 000 children tested at ages 1, 4, 8, 12, 16 and 19 years of age; Group C: 25 000 children tested at 1.5, 5, 9, 13, 17 and 20 years of age; Group D: 25 000 children tested at 2, 6, 10, 14, 18 and 20 years of age. It is noteworthy that this approach can also be used with other testing strategies in mind. For example, if all study population children were to be tested at fixed ages, the set of tests recommended for each designated age could be administered. The reader is also referred to the NICHD website (http://www.nationalchildrensstudy.gov/research/analytic_reports) which includes other details about the tests described in this section. The rationale for choosing the tests for each domain will be reviewed, along with a summary of the advantages and disadvantages of each test for both recommended and alternative tests. A brief section will follow describing special requirements of test administration during each of the six proposed age ranges and screening possibilities during some testing cycles.

### Assessment domains by age omnibus intelligence (IQ) and abilities measures

These measures assess potential exposure effects on omnibus measures of general mental abilities at ages that have proved to be critical in previous studies (0–2 and 3–6 years), ages at which such measures are relatively stable and should reflect IQ across childhood (7–10 years), and at an age when long-term effects on IQ of earlier exposures can be evaluated (15–18 years).

For age 0.5–2 years, the Bayley Scales of Infant Development-II^7^ are recommended. Although other scales exist (eg, Fagan test, Brazelton Scale), the Bayley Scale has the best standardisation and has been used extensively in previous exposure-outcome research. For example, it has been applied to assess the effects of lead,^8^^–^12 PCBs,^13^^–^15 methylmercury^16^^–^18 and dichlorodiphenyldichloroethylene.^19^ ^20^ Although the Bayley Scale is a fairly blunt instrument that may not pick up subtle deficits associated with exposures and the items are rather diverse (ie, they do not easily lend themselves to domain-specific analysis), it is the best option available. It is recommended that this test be given to the cohort subgroups at one time point each (6 months, 1 year, 1.5 years or 2 years).

At ages 3–6 years an abbreviated version of the Wechsler Preschool and Primary Scale of Intelligence (WPPSI-III)^21^ (Block Designs, Matrix Reasoning and Vocabulary) is recommended. The WPPSI-III was chosen over other possibilities for several reasons. The Wechsler scales have been the most extensively applied to IQ research on this age range in the past and they dovetail nicely with Wechsler subtests available at later ages. The subtests chosen will produce an IQ score, have parallel versions available at later ages and can contribute some information to domain-specific function (although they are far from pure measures of specific domains). The major disadvantage of the Wechsler Scales is that they have somewhat abbreviated ranges, meaning that at the lowest and highest age ranges the tests can be too difficult or too simple and that persons with low IQs or those who are gifted may be “out of range” (ie, unable to meaningfully complete subtests or able to correctly complete all or virtually all items).

Other tests that were considered for this age span include the McCarthy,^22^ which has also been used extensively in developmental research. The major disadvantage of this test is the limited number and heterogeneity of items in specific subscale areas. The Stanford Binet Intelligence Scale-V^23^ was also considered. The major advantage of this test is that it can be given across the lifespan, frequently using the same subtests. It is also excellent for measuring the higher and lower ends of intelligence. Disadvantages include limited use in developmental research, less information on relationships between subtest performance and developmental outcomes, and the fact that there is limited experience with the most recent edition of the scale. The publishers have significantly altered the test; an important omission from the new version appears to be the copying test, which proved to be very sensitive in several cultures at several ages to the effects of prenatal and childhood exposure to methylmercury. Finally, the Kaufman scales (KABC-2, KBIT-2)^24^ ^25^ were considered. These scales have also been used less extensively in developmental research and have recently undergone significant revision, raising questions about their comparability to previous versions of the tests. The Raven Progressive Matrices Test^26^ has been used in the past in toxicant exposure studies. This test has been successfully applied in many cultures and appears to possess inherently less cultural and linguistic bias than other intelligence tests. However, the Raven test assesses intelligence in a one-dimensional fashion (a type of non-verbal executive function) and supportive psychometric data for the test are limited with regard to norms, validity and reliability.

At ages 7–10 and 15–18 years the Wechsler Abbreviated Scale of Intelligence (WASI)^27^ is recommended to assess the general abilities domain. This test has four subtests (Block Designs, Matrix Reasoning, Similarities and Vocabulary) that provide continuity with the WPPSI-III subtests recommended for children aged 3–6 years. In addition, the WASI can be used across the lifespan after age 6 using the same subtests. Another possibility for children aged 7–10 years is the Wechsler Intelligence Scale for Children (WISC)-IV,^28^ a revised version of the earlier WISC scales (WISC, WISC-R, WISC-III) which have been used extensively in developmental research. The WASI was recommended over the WISC-IV owing to its greater brevity and its continuity across the developmental span.

For the 15–18 age range, inclusion of a full Wechsler assessment would require use of both the WISC-IV and the WAIS-III, switching tests at age 16 or 17 years. This would produce less continuity in the age range testing, and both scales can be problematic for 16-year-old subjects (too easy or too hard).

The NEPSY^29^ was considered as an alternative test for both omnibus scores and domain-specific assessment and is seen as an alternative instrument. The test seems to have a rather low ceiling and subtest length is somewhat limited, restricting the utility of outcome data.

When discussing the neuropsychological domains below, subtests from the IQ tests and the NEPSY can always be considered to be possible alternative tasks. The pros and cons of these tests have been described above and will not be repeated.

### Academic screening

A domain for brief academic testing was included as an assist to screening for learning disorders. This domain was not designed to serve as a full assessment of academic abilities as outcome measures, although the results can be used as a cursory evaluation of these outcomes at the age ranges in which they are included. In children aged 7–10 years, brief testing of basic academic skills can be combined with results of IQ and domain-specific testing in order to identify those who may have disorders of learning. The test recommended for this domain is the Wide Range Achievement Test-4 (WRAT-4),^30^ which assesses single-word reading, single-word spelling and arithmetic. This test was selected for ease of administration, time efficiency and its acceptance in the field. It has seen limited use in exposure studies. Alternative tests include the Woodcock-Johnson^31^ which was used in a study of methylmercury, the Kaufman Test of Individual Achievement-2 and the Wechsler Individual Achievement Test.^32^ These tests are more complex than the WRAT-4 and less suited to screening. It is recommended that the WRAT-4 be repeated during the 15–18 age testing in order to assess stability of any exposure-related changes in basic academic skills over time.

### Attention/concentration

The cognitive processes subsumed under this domain have been widely described and evaluated in the cognitive psychology literature. Since it is not possible to assess all aspects of the domain, this assessment strategy focuses on behaviour, sustained attention/reaction time and spans of apprehension.

Tests recommended for this domain among 3–6-year-old children include the Conners Rating Scale-Revised^33^ and the Conners Continuous Performance Test (CPT)-II.^34^ The Conners Rating Scale is used to assess behavioural characteristics that are associated with attention deficit hyperactivity disorder (ADHD) as defined by the Diagnostic and Statistical Manual-IV (DSM-IV). Outcomes include both a score that can be used as a quantitative outcome measure and assignment of a provisional diagnosis of ADHD based on cut-off criteria. Thus, the test can contribute to screening for ADHD in this age range. Testing with the Conners CPT-II begins at age 6, and the test allows evaluation of lapses in attention (omission errors), over-responding (false positives) and reaction time. Reaction times have proved to be sensitive indicators of exposures to toxicants and medications. The Conners CPT-II was recommended because of its widespread use in child clinical neuropsychology. Span of apprehension testing (Wechsler Digit Span Forward) is not recommended for this age group owing to limited applicability at the age of 3–5 years.

For children aged 7–10 years, it is recommended that the Conners Rating Scale-Revised be repeated in order to acquire a second set of outcome scores on attentional behaviours and to allow a second chance to pick up possible cases of ADHD that were missed at previous testing. The Conners CPT-II is also recommended at all ages due to the sensitivity of reaction time data to many types of exposures/insults/disorders. Finally, the Wechsler (WISC-IV) Digit Span Forward test is recommended as a span of apprehension task. This task provides data on the number of bits of information that the child can automatically register and repeat back. Such data are important as outcome measures (and have been related to specific types of exposures during development). They can also be used to estimate appropriate expectations for performance on learning tests. Other possibilities include the Neurobehavioral Evaluation System (NES) letter or animal CPT,^35^ which has also been used extensively and effectively in detecting subtle toxicant effects in children and adults. The NES is less widely available and more difficult to adapt to different testing situations than the Conners test. Normative and psychometric data are also less extensive for it. A visual pointing span test is another alternative.

For children aged 11–14 years, it is recommended that the Conners CPT-II and the WISC-IV Digit Span Forward be repeated. At age 14 the Conners Rating Scale-R can be repeated to assess stability of scores and cut-offs for ADHD diagnosis criteria.

At ages 15–18, it is recommended that the Conners CPT-II and Wechsler Digit Span Forward assessment be repeated (WISC-IV for ages 15–16; WAIS-III for ages 17–18). The Conners Rating Scale-R can be repeated at ages 15, 16 and 17 years.

At ages 19–20 the recommended attention test for the brief battery is the Conners CPT-II.

### Executive function/working memory

This domain is a complex one and related skills tend to develop somewhat later than those subsumed under other domains. Since it would be impossible to evaluate all aspects of this domain at every age level, testing is limited to screening for a few skills at each age level. The tests chosen for the age ranges between 3 and 18 years were selected to include both visually and verbally mediated tasks.

Proposed testing begins in the 7–10 year age range. The Wisconsin Card Sorting Test (WCST)^36^ is a widely used task assessing this domain. It taps inferential reasoning, working memory, capacity to attain and switch sets flexibly, and ability to inhibit distractions and perseverative tendencies. Performance on this test has been related to a wide variety of neurological and developmental disorders, neuropsychiatric syndromes and exposures to chemical toxicants. A second test, the WASI Similarities test, is administered as part of the general intelligence testing and also taps aspects of executive function. This task assesses abstract reasoning and its performance scores have been related to aspects of normal and abnormal child development. The Children’s Categories Test^37^ is an alternative test for the same domain. It has two levels (for ages 5–8 and 9–13 years), so the test stimuli are different for children tested at ages 7 and 8 years than for those tested at 9 and 10 years. A few of the items are slightly problematic. Other tests that assess this domain that can be considered as alternatives for use in the 7–10 year range include the Children’s Color Trails Test^38^ (age 8–10 years) and the Stroop Color Word Test.^39^ The Color Trails Test appears to be relatively culture fair but has (in RFW’s experience) been difficult to administer (many children do not understand it initially). There is also much less information about how performance on this test relates to other aspects of childhood cognitive development than is available for other tests of executive function. The Stroop Test is commonly used as an executive test and much more is known about its relationship to other variables. However, performance on the test varies widely, affecting reliability and psychometrics of the test for data analysis purposes.

The executive domain tests recommended in table 1 for the 11–14 age group are Wechsler (WISC-IV) Digit Span Backward, a working memory task requiring the registration and manipulation of verbal information, and the Trail Making Test (TMT)^40^ which requires the examinee to track and connect visual information (A condition) and to alternate sets while tracking and connecting visual stimuli (B condition). Both tests have rich sources of scientific and clinical data to support their use and interpretation in a study such as this. Drawbacks to the Digit Span Backward include resistance to the task by examinees who feel they cannot manipulate numbers as well as the need for considerable examinee cooperation in completing it. The TMT is a timed task and optimal performance is only elicited when the examinee is willing to work as quickly and accurately as possible. It also requires automatic knowledge of the alphabet sequence and numbers. Alternative tests include the tests described above for examining this domain in children aged 7–10 years.

For testing in the 15–18-year age group, it is recommended that Wechsler Digit Span Backward (WISC-IV for age 15–16 years, WAIS-III for age 17–18 years), the WCST and WASI Similarities be repeated. Other possibilities include the Children’s Categories Test (Level 2),^37^ Children’s Color Trails^38^ (1–17) and Color Trails^41^ (age 18 years). Pros and cons of these tests have been noted above. Another alternative is the Paced Auditory Serial Addition Test (PASAT),^42^ a rather difficult task that was developed to demonstrate subtle brain damage associated with head injuries. A strength of this test is its sensitivity to subtle processing deficits. However, because it requires considerable examinee cooperation, performances tend to be rather variable. It is not as well represented in the general developmental cognitive literature as some of the other tests mentioned.

The recommended executive domain task for the brief testing of subjects aged 19–20 years is the TMT, repeated 7–8 years after initial presentation to the cohort. This is a highly sensitive and efficient test that is also well investigated and appropriate for young adults.

### Language/verbal skills

Goals for assessment of this domain include examination of lexical knowledge, simple verbal comprehension and ability to define vocabulary words while allowing preliminary screening for speech disorders and verbally-based learning disabilities at the younger ages. The group of tests recommended for each age range includes tasks assessing both expressive and receptive aspects of language skills.

During the assessment of children aged 3–6 years it is recommended that simple naming of objects be evaluated. Both tests recommended for the assessment of naming require the child to name objects presented in drawings or pictures. The naming portion of the Expressive Vocabulary Test (EVT)^43^ is recommended for children aged 3–5 years. The EVT was chosen because of its applicability for expressive language during childhood and the particular balance of naming/synonyms in assessment of language function over development. At 6 years of age a different naming test must be applied. The Boston Naming Test (BNT)^44^ is recommended owing to its feasibility from the age of 6 years through adulthood and its known effectiveness in detecting subtle effects of prenatal exposure to toxicants such as methylmercury. It has been applied in widely diverse cultures and subcultures and translated into many languages. Another possible test that could be used to evaluate naming in this age range is the WPPSI-III Naming Test which can be administered at all four ages (3–6 years), although it does not have a parallel version for use at later ages.

The Peabody Picture Vocabulary Test-III (PPVT-III)^45^ is recommended as a test of receptive language or language comprehension for this age range. It has a number of advantages, including ease of administration, well-documented reliability and validity, extensive norms, well-defined psychometrics and widespread use in the field. Similarly, the Token Test for Children^46^ has normalised scores for ages 3–12 (in 6-month increments). It was developed as a rapid screening measure of language competence, particularly for children with receptive language dysfunction that depresses language scores. Both tests are appropriate but the Token Test was selected as an alternative to the preferred PPVT because it is less process-specific in its task demands.

It is recommended that assessment of ability to provide definitions of words be carried out with the WPPSI-III Vocabulary Test, a test with the same advantages as PPVT-III. Another assessment, the Clinical Evaluation of Language Fundamentals-IV (CELF-4)^47^ includes both expressive and receptive subtests, one of which is also recommended for use at a later age level. However, most of the CELF-4 subtests appear to be less appropriate for screening in the 3–6-year age group than those included in the recommended list.

At ages 7–10 years the BNT is recommended as an assessment tool for expression/naming, the PPVT-III for comprehension/receptive speech and the WASI Vocabulary subtest for production of word definitions. Pros and cons of these tests and alternative tasks are noted above.

Recommended tests for the language domain in the 11–14-year age group include two new tasks that allow examination of a slightly different aspect of expressive and receptive processing and thus reduce practice effects across the age ranges. The EVT Synonyms task (requiring the examinee to produce words with the same meaning as those given orally by the examiner for a stimulus picture) and the CELF-4 Sentence Structure subtest (a receptive task requiring recognition of words appropriate to sentences) are recommended for this age range. These tasks are less well known than the language tests recommended for earlier age ranges but have been well standardised and normed and will provide information on more complex aspects of expressive and receptive language at this age level.

For the language/verbal assessment at ages 15–18 years, it is recommended that the BNT, PPVT-III and WASI Vocabulary subtest be repeated.

The recommended brief language domain assessment at ages 19–20 years includes repetition of the EVT (Synonyms) and CELF-4 Sentence Structure subtest. It is also possible to repeat the BNT or PPVT-III or to apply other language tasks.

### Visuospatial abilities

The critical processes that must be evaluated in the assessment of visuospatial abilities have been less well defined than those of other domains, and there has been considerable overlap in the stimuli used across visuospatial tasks designed for children. To accommodate potential individual differences, it was deemed important to include both traditional constructional tasks (with a motor component such as drawing or putting blocks or puzzle pieces together) and motor-free tasks that involve visuospatial processing and integration at a cognitive level only.

The visuospatial tasks recommended for use at ages 3–6 years include the Visual Motor Integration Test (VMI-5).^48^ This task is well embedded in the developmental literature and has recently been revised and renormed. It has been used in previous work involving environmental toxicant exposure and was chosen partially for its similarity to the Copying Test of the Stanford Binet-IV.^23^ The latter task was highly feasible in several cultures and able to detect subtle effects of early exposures to methylmercury. The Copying Test was also valuable because it could be administered across the lifespan, a property not associated with the VMI-5. However, the two tests have overlapping stimuli and test requirements. An alternative constructional test is the Bender Gestalt-II.^49^ The original version of this test has been used extensively in both clinical and research situations and has detected effects of toxicant exposures. However, for this age range the test could only be administered at ages 5 and 6 years. The WPPSI-III^21^ Block Designs and Matrix Reasoning subtests are recommended for the 3–6-year age range. They provide a measure of visuospatial skills with a motor component (Block Designs) and a test without motor requirements (Matrix Reasoning). Both have a strong executive component (as do many visuospatial tests). They also contribute to the IQ score recommended for this age group.

The Block Designs and Matrix Reasoning subtests of the WASI are recommended for the 7–10-year age range. Advantages and drawbacks are similar to those described for the subtests at ages 3–6 years, though the WASI subtests can be given across the lifespan after age 6. A second recommended visuospatial task for children aged 7–10 years is the Bender Gestalt-II, a visual constructional task similar to the initial version used in prior research but with additional designs added for a better range of scores and difficulty level. Experience with the revised version is still somewhat limited. It has a recall condition, an advantage that contributes to the test’s efficiency in a battery such as that to be used for the National Children’s Study.

For children aged 11–14 years, three tests are recommended. Repetition of the VMI-5 is suggested as a measure of visual constructions. The Hooper Visual Organization Test (HVOT)^50^ is recommended as a motor-free task assessing visual integration. The test has a somewhat low ceiling in adults, but is one of the few tests available that allows examination of visual organisation without drawing or assembling concrete objects. Finally, the Rey-Osterreith Complex Figure (ROCF) test^51^ is recommended for use in this age range. The ROCF is a relatively difficult construction that increases the range of assessment of the visuospatial domain, can be given quickly, is well known to clinicians and researchers in child development and includes memory conditions. Scoring of the ROCF can be done simply, although complex scoring systems have been developed for it. This task also produces a wealth of qualitative information that may be useful for certain kinds of data analysis.

The visuospatial tasks recommended for the 15–18-year age group assessments include the Bender Gestalt-II and two WASI subtests (Block Designs and Matrix Reasoning).

For the 19–20-year age group the brief visuospatial battery recommended includes the motor-free HVOT and the ROCF to assess constructional ability.

### Learning and memory

As noted in the definitions above, learning and memory function involve several key cognitive processes. Luckily, several tests of learning and memory have been developed that address all or most of these functional processes. For the recommended battery, this domain is focused on anterograde memory rather than retrograde memory or procedural learning. Because children differ in their verbal and visuospatial abilities and because the cerebral structures subserving the processing of verbal and visual information are different, visual and verbal memory tests are included at each age level. Similarly, within the verbal modality, learning lists of words or word-pairs can be differentiated on a neural system or neurofunctional basis from learning discourse or paragraph material. For this reason, both word list and discourse tasks were included as much as possible at each age level.

The battery for this domain is relatively limited in the 3–6-year age range. Few tests are available and administration of these kinds of tests is difficult for very young children. The recommended test battery takes advantage of the use of the WPPSI-III Coding Test at ages 4–6 years (see Motor domain below) to carry out incidental learning of the symbol-symbol pairs (visual memory task). At 5–6 years of age, administration of the California Verbal Learning Test (Children’s version) (CVLT-C)^52^ is recommended. This is a list-learning test devised to comprehensively assess learning and memory at several levels, with learning, immediate and delayed recall conditions as well as spontaneous and recognition test paradigms. It is thus a rich test that provides considerable information. It is somewhat time-consuming and some individuals resist list-learning tests, but its advantages were judged to outweigh disadvantages. The Wide Range Assessment of Memory and Learning-2 (WRAML-2)^53^ Stories subtest is also recommended for children aged 5–6 years. The previous version of the WRAML has been used in research of this type and appears to be solid with regard to psychometrics and standardisation. It was recently revised, but a review of the test suggests that the recommended subtests have not significantly changed.

At 7–10 years of age the breadth of testing in this domain widens considerably. Recommended tests for the assessment of visual learning and memory include the recall condition of the Bender Gestalt-II and incidental learning of the visual pairs from the Wechsler-IV Coding subtest. For verbal learning the CVLT-C list-learning test and WRAML-2 Stories test are again recommended (stimuli for the WRAML-2 Stories test change at age 9 years).

Recommended visual memory tasks at 11–14 years of age include the recall condition of the ROCF and incidental recall of the pairs from the WISC-IV coding tests. Verbal memory tests included at this age range in the recommended battery include the WRAML-2 Stories subtest and the WRAML-2 Verbal Learning subtest (a list-learning task).

It is recommended that visual memory for 15–18-year-olds be assessed using the immediate and delayed recall conditions of the Bender Gestalt-II and incidental recall from the Wechsler Coding subtest (WISC-IV at age 15–16 years, WAIS-III at age 17–18 years). Assessment of verbal list learning using the adult version of the California Verbal Learning Test II (CVLT-II) is recommended. For narrative or discourse learning, repetition of the WRAML-2 Stories subtest is suggested for children aged 15–16 years and the Logical Memory subtest of the Wechsler Memory Scale-III (WMS-III) for those aged 16–17 years.

The recommended brief battery for assessment of learning and memory at ages 19–20 includes the ROCF immediate and delayed recall, CVLT-II and WMS-III Logical Memory.^54^

### Motor skills

There are few quantified fine motor tests for children or adults. In designing the batteries to assess motor function, the decision was made to recommend a simple test of motor speed, a more complex test that invokes both speed and dexterity and an integrative task. All recommended tests evaluate manual motor speed with the hands, with at least one test at each age level allowing comparison of the right and left hands.

The recommended assessment of motor function at ages 3–6 years includes the Revised Purdue Pegboard.^55^ This test is a local adaptation and test instruments may have to be constructed for assessing this domain in small children. At age 4, administration of the Wechsler Coding subtest begins (using the WPPSI-III subtest). This task is highly sensitive to many brain insults and developmental conditions but is not specific with regard to localisation or diagnosis. The test itself is completed manually and timed, but performance improves if the child uses an effective strategy, has strong visual orientation and/or visual scanning skills and can remember the stimuli. Thus, the task requires integration of many abilities. However, its inclusion is recommended based on its sensitivity. An incidental memory condition can be used to enhance the assessment of the memory domain as well (see above).

The recommended group of tests for evaluating this domain is the same from ages 11 to 20 years. It includes the Fingertapping Test^56^ for a simple assessment of manual motor speed with each hand. Computerised versions provide especially precise data on Fingertapping and a system such as the Neurobehavioral Evaluation System (NES) might be used, but it is important that the testing be the same at all test sites. The mechanical and automated tappers are standard, but sometimes malfunction and may produce different data at different sites. Alternatives include the NEPSY Fingertapping Task (done with the examinee’s fingers), but this test is much less precise. The Grooved Pegboard^57^ is recommended to assess motor speed involving dexterity/coordination with each hand. A standard form board can be purchased. This test is widely used. It is recommended over the Purdue Pegboard because it picks up more subtle brain damage. The Santa Ana Formboard^58^ has a special place in the neurotoxicology literature and, like the Grooved Pegboard, is more challenging than the Purdue. Standard versions of it are not available, although they could be constructed.

### Other categories

#### Novel tests

This category was included in the matrix in order to indicate propitious times at which study participants could undergo other kinds of tests when they had not been tested for a while. Thus, children from Group A who are tested at age 3 and not scheduled for standard evaluation again until age 7 might undergo another test battery at age 5. Applicable scenarios are described below.

#### Pilot testing

Tasks to be introduced later or tests that are revised could be piloted at these time intervals.

#### Evaluation of domains not included in the standard battery

Test batteries to assess domains such as prosody (expression, comprehension), tactile and kinesthetic functions, retrograde memory and procedural learning might be applied as deemed appropriate or useful. In addition, a test of purposeful test failure was not included since there does not seem to be motivation for cohort members to fail tasks. However, under certain circumstances, the introduction of such a test might be warranted.

#### Animal test techniques

Tests adapted from animal studies could also be applied and piloted. These might include existing operant tasks and tests such as the Delayed Recognition Span Test^59^ (a learning task based on delayed non-matching to sample methodology).

#### Special studies

Substudies assessing special abilities or tasks exploring the cognitive processes thought to subserve domains might be applied at these times.

### Age ranges

As noted in table 1, the six age ranges were selected to allow testing of each child every 4 years from age 3–6 to 15–18 years, with additional testing at age 0.5–2.0 and 19–20 years. It is recommended that the age band before and after the target age be restricted as follows: ±1 month at age 0.5–2.0 years and +0–3 months at ages 3–20 years.

### Screening considerations

Test performance, especially at earlier ages, can be used as a signal that further evaluation is necessary for possible disorders or illnesses. At age 0.5–2.0 years, Bayley-II results could be used to tag children for evaluation of mental retardation (>2 standard deviations below average on the Mental Development Index), cerebral palsy or motor disorders (>2 standard deviations below average on the Psychomotor Development Index) or autism (<10 percentile on the behaviour rating scale).

In children aged 3–6 years, Conners Rating Scale-R scores below the cut-off point can tag children for evaluation of ADHD. Children scoring >2 standard deviations below average on the Conners CPT-II or making large numbers of errors can be considered for evaluation of other types of disorders (eg, autism, neurological conditions such as epilepsy). Individuals with IQs below 70 on the WPPSI-III can be tagged for possible evaluation of mental retardation. Large discrepancies between verbal and visuospatial skills on the WPSSI-III subtests accompanied by similar discrepancies on the domain-specific tests could be used to identify children at risk for speech disorders, learning disabilities, neurological disorders or autism.

In children aged 7–10 years, those with borderline intelligence or those who are gifted can be identified using WASI data. Children with large discrepancies between verbal and performance measures on the WASI who also show large discrepancies between reading and arithmetic on the WRAT-3 can be considered for evaluation of possible learning disorders.

### Other considerations for test implementation and review

The following suggestions apply to implementation and data collection of the neuropsychological data in future studies.

#### Pilot work

All tests should be piloted on a representative sample of children before they are given to each group in order to confirm that time allotted for the battery is accurate in the population being assessed, to determine the best order of tests administered and to identify feasibility issues such as acceptability to examinees, variability in test administration among examiners and cultural/language aspects of the tests that may not have been anticipated.

**What is already known on this subject**

- Over the past 25 years, several neuropsychological test batteries have been proposed for the assessment of central nervous system (CNS) effects of environmental exposures on CNS function in children.
- These have largely employed traditional tests of intelligence quotient (IQ) or academic skills, although a few have taken a broader approach including domain-specific tests of cognitive, motor and affective function.

#### Revised versions of tests

Virtually all of the tests included in a planned battery will be revised over time, sometimes changing the task demands and stimuli quite significantly. If this is done without the necessary psychometric work, piloting of new tests will be required to evaluate their comparability to the task already in use. It may be necessary to decide whether to continue to use an “old” version of a test or to implement the revised version. Obviously, continued use of the older version will enhance comparability of raw test scores across development. However, revised versions of tests may be more useful for reporting or may better fit new theories or knowledge about brain-behaviour relationships.

**What this study adds**

- A test battery aimed at assessing the effects of environmental insults on CNS function that is longitudinal and cohort-focused.
- A wide-ranging test battery, including tests of IQ as well as tasks that assess specific cognitive, affective and motor domains.
- A test battery that employs a strategy of dividing a cohort into groups so that, over time, all domains are tested in all age groups from age 0.5–20 years.
- A test battery that uses a rationale for the tests chosen that includes developmental appropriateness of domains assessed and specific tests in each age range, including tasks that can be repeated across time to determine changes in actual raw scores on tests as children develop while at the same time introducing new tests when necessary.
- A test battery that includes tests that have detected effects of exposures to neurotoxic chemicals in past research, while at the same time allowing for hypothesis-driven research that explores the structural and functional underpinnings of these effects.

#### Adapting tests

Tests and test scoring rules may not apply to specific cultural or linguistic groups. Care will be needed to address these issues within the cohort, again with pilot testing, review of protocols with regard to scoring and translation or development of language- or culture-appropriate tests for certain groups of children.

This document has noted that some children will have special kinds of cognitive disorders that affect their performance. There will be some children who cannot complete even the lowest levels of the tasks recommended in the present battery. For children who are identified with extremes in IQ (especially children with borderline IQ or mental retardation but possibly also children with very superior intelligence), it may be necessary to develop specialised batteries for assessing domain-specific functions. Children with learning disabilities or ADHD but normal intelligence can be presented with the battery tasks, although of course their disabilities will affect performance levels and types of errors.

Sensory deficits may also present difficulties. Tasks can be adjusted for children who cannot see or hear or who have motor deficits. These adjustments should be standardised for the cohort of children and noted in data summaries.

#### Data collection

Verbatim recording of examinee responses and the use of answer sheets that allow the examiner to record approaches to tasks and other aspects of performance will enhance the data set. Generally, raw scores are most useful in studies of these kinds for data analysis, although conversion to normative scores may be useful for some purposes. It is generally impossible to record all qualitative data in databases, although the most relevant should be determined and included (eg, number of phonemic errors on a naming test). When raw data are carefully collected, qualitative findings can be reviewed and summarised later if specific questions arise.

#### Examiners

Educational requirements for examiners should be carefully considered. Licensed doctoral level psychologists with training in assessment might be preferred since they are more likely to respect standardisation rules or note possible disorders in children as they are testing them. However, supervised master’s level licensed psychometrists are an option as are highly trained bachelor’s level research assistants. Extensive training and monitoring of examiners will be necessary, together with evaluation of intra- and inter-examiner reliability of examiners. Videotaping of testing is recommended.

#### Examinee effort

Effort on the part of the examinee will affect scores. It may be useful to employ brief rating scales for indications of effort to be filled out by both the examiner and the examinee.
